# In-bag extraction of tissue through an incision in the posterior vaginal wall in laparoscopic myomectomy: a large retrospective study

**DOI:** 10.1186/s12905-023-02780-8

**Published:** 2023-11-27

**Authors:** Xin Zhao, Yansong Liu, Yulin Shi, Jumin Niu

**Affiliations:** Shenyang Women’s and Children’s Hospital, Shenyang, Liaoning Province China

**Keywords:** Laparoscopic myomectomy, The middle part incision of posterior vagina, In-bag tissue extraction, Surgical specimen retrieval

## Abstract

**Background:**

Our purpose was to describe the outcomes of transvaginal in-bag tissue extraction tissue through an incision in the posterior vaginal wall the middle part incision of posterior vagina in laparoscopic myomectomy.

**Methods:**

This was a retrospective study of patients who received laparoscopic myomectomy and in-bag tissue extraction through an incision in the posterior vaginal wall between January 2016 and December 2022. Patient characteristics, intra- and post-operative complications, and outcomes were collected and analyzed.

**Results:**

A total of 511women were included in the analysis. The mean largest myoma diameter was 8.44 ± 3.56 cm; mean specimen weight was 789.23 ± 276.97 g; mean operative time was 129.01 ± 53.13minutes; and mean blood loss was 175.99 ± 210.96 mL. Within 30-days of surgery, no fever, infection, or vaginal bleeding was noted in any patient, and the vaginal incisions of all patients had healed well. There were no incisional hernias, pelvic infections, and vaginal adhesions noted at follow-up 3 months after the operation. There were 37 cases of vaginal delivery of the patients after surgery, and there were no lacerations of the posterior wall vaginal incision.

**Conclusions:**

Transvaginal in-bag extraction though an incision in the posterior vaginal wall is feasible and safe for removing tissue after laparoscopic myomectomy.

## Introduction

Myomas are the most common benign tumors of the genital organs in women of childbearing age [1]. In many women, they can cause marked morbidity and impairment of quality of life. It is estimated that myoma are clinically apparent in 25% of women of reproductive age, and in 25% of women cause symptoms severe enough to require treatment [2–4]. Myoma may be asymptomatic, or cause a range of severe and chronic symptoms. The most common presenting symptom is heavy menstrual bleeding, which can lead to anemia [5, 6]. Other myoma symptoms include non-cyclic pain, painful intercourse or pelvic pressure, and bladder or bowel dysfunction resulting in urinary incontinence or retention, pain or constipation [5–7]. The presence of large intramural and submucosal myomas has been associated with infertility and increased risk of spontaneous abortion, fetal malpresentation, placenta previa, preterm birth, and Cesarean delivery [8–10].

Myomectomy is the first choice of treatment for patients who wish to retain fertility, or are unwilling to undergo a hysterectomy. The surgical methods include vaginal myomectomy, open abdominal myomectomy, or laparoscopic myomectomy. Laparoscopic myomectomy is the most commonly used surgical method, and is minimally invasive, is associated with a short hospital stay and rapid recovery, and is cosmetically pleasing [11, 12]. However, in 2014 the US Food and Drug Administration (FDA) warned about the risk of inadvertently disseminating undetected leiomyosarcomas when using uncontained laparoscopic power morcellation for myoma removal [13]. Since then, power morcellation for myoma removal during laparoscopic myomectomy has been avoided.

There are 4 main methods for specimen extraction when performing laparoscopic myomectomy: (1) a small abdominal incision, (2) enlarging the umbilical incision to remove the specimen, (3) placing a protective bag in the abdominal cavity, placing the myoma in the bag, and then cutting/crushing the myoma after it is in the bag, (4) removing the myoma through an incision in the posterior fornix of the vagina [14, 15].

In our practice, we make an incision in the middle section of the posterior wall of the vagina, place the extraction bag into the abdominal cavity through the vaginal incision, put the myoma into the bag, and then remove the bag with the myoma through the vaginal incision. We have found the method to be a feasible and cost-effective method that avoids expanding the skin incision, and eliminates the need for power morcellation and the potential spread of malignant cells.

Thus, the purpose of this study is to report our experience with this method over the past 7 years.

## Materials and methods

### Patients, preoperative work-up, and data collection

The records of 511 patients with myoma who underwent laparoscopic myomectomy with in-bag tissue extraction through an incision in the middle of the posterior vagina at Shenyang Women’s and Children’s Hospital between January 2016 and December 2022 were retrospectively reviewed. Data were retrieved from our institution’s surgical database, which was developed for research purposes.

Inclusion criteria were: (1) Myomectomy for single or multiple symptomatic uterine myomas; (2) Surgery performed by a laparoscopic approach; and (3) In-bag tissue extraction through an incision in the middle part of the posterior vagina was performed. Exclusion criteria were: (1) Malignancy suspected during the preoperative work-up; (2) Not scheduled for transvaginal specimen extraction to preserve the integrity of the hymen; and (3) Obliterated Douglas pouch (e.g., deep infiltrating endometriosis of the posterior compartment). Before surgery, all patients were counseled that the specimen would be removed transvaginally. All patients provided written informed consent for laparoscopic myomectomy and in-bag transvaginal extraction through an incision in the middle of the posterior vagina, and informed consents met the requirements of the hospital ethics committee.

In all patients, uterine myoma were diagnosed by pelvic ultrasound and/or pelvic magnetic resonance imaging (MRI). All the operations were performed by gynecologic surgeons with extensive experience in advanced minimally invasive surgical techniques (> 500 major gynecologic laparoscopic procedures per surgeon).

Data extracted from the medical records included age, body mass index (BMI), parity, main indication for the myomectomy, previous pelvic surgery, single or multiple myoma, diameter of the largest myoma removed (as measured on preoperative ultrasound/MRI), surgical specimen weight, operative time (defined as the time from the initial skin incision to when the last skin stich was applied), time needed to remove myoma, vaginal incision suture time, estimated blood loss (calculated at the end of surgery from the contents of the suction devices and reported in mL), length of hospital stay, intraoperative and perioperative complications (including estimated blood loss > 500 mL), and complications occurring within 30 days of surgery. Patients were advised to avoid intercourses during the first 30 days after surgery to allow proper healing of the incision in the posterior vaginal wall.

### Laparoscopic myomectomy

The anesthesiology protocol was standardized, and all patients received general anesthesia with endotracheal intubation. Patients were placed in the prone position at a 30° angle (head low, feet high). All myomectomy were performed in a standard manner.

In brief, a multiport laparoscopic technique was used. A 10 mm incision is made at the upper edge of the umbilicus to establish a pneumoperitoneum, and 3, 5 mm Trocar were placed in the left and right side of the abdomen. For subserosal myomas, bipolar coagulation is used to remove the root of the myoma, and to stop bleeding. If necessary, sutures are placed to assure hemostasis. For intramural myomas, a monopolar electric hook is used to cut the myoma pseudocapsule, separate the pseudocapsule, and expose interior of the myoma. The myoma is then grasped with a 5 mm grasping pliers, and peeled off. A 1 − 0 absorbable suture is placed continuously to close the seromuscular layer using a single- or multi-layered closure technique. Anti-adhesive barriers and hemostatic agents are not routinely used.

### Vaginal posterior wall incision

**Fig. 1:**
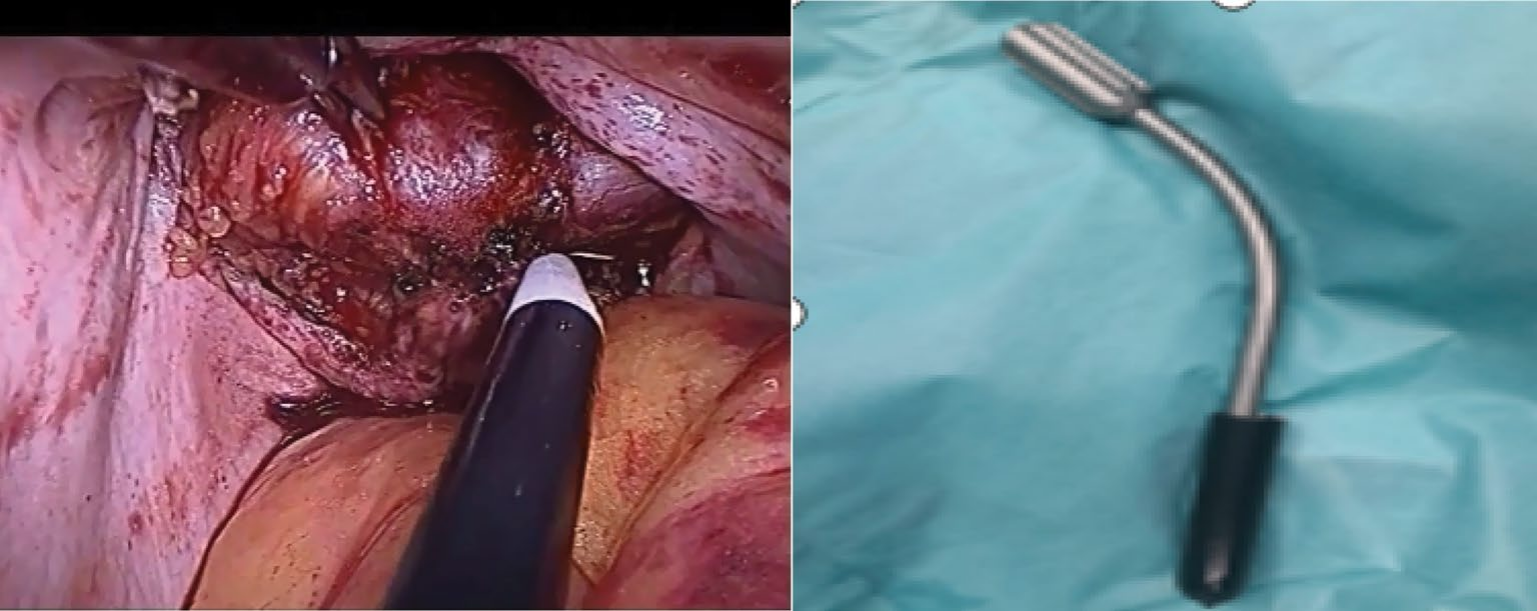
Vaginal separator used to release the posterior wall of the vagina

**Fig. 2:**
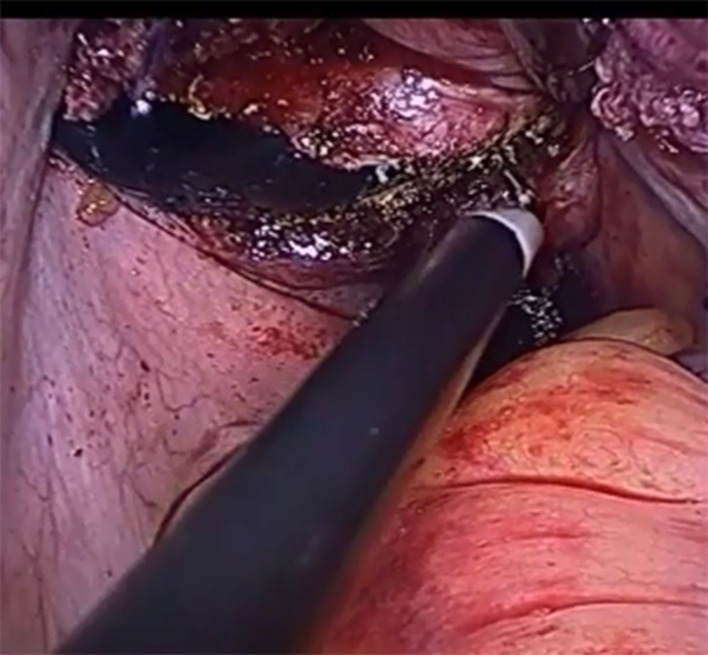
Monopolar hook is used to cut the posterior wall of the vagina between the sacral ligaments

**Fig. 3:**
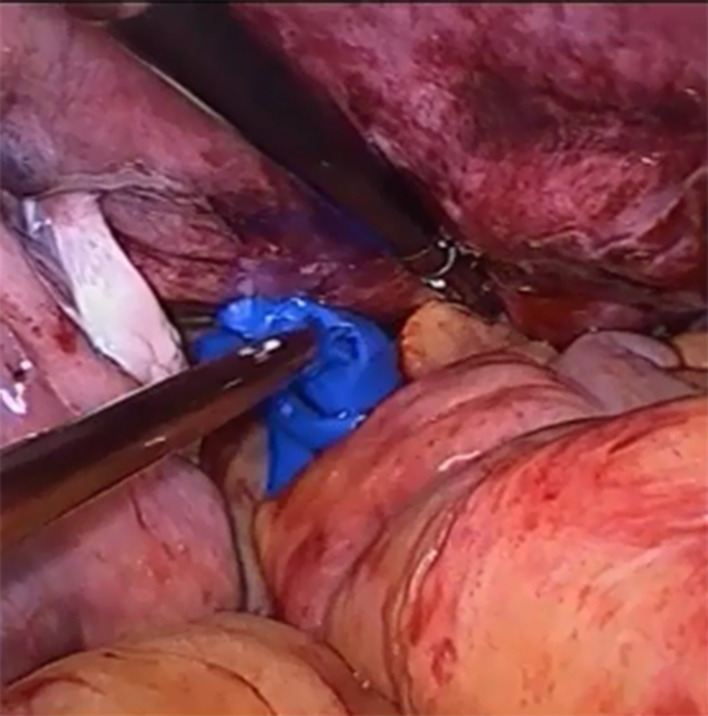
Extraction bag is placed into the abdominal cavity through the vaginal incision

After completion of myoma removal, the Douglas cavity is exposed, the rectum is retracted, and the peritoneal junction between the rectum and the vaginal wall is identified. If the peritoneum is clearly identified, it is incised and the incision is extended to both sides with attention given to the depth of the incision. If the peritoneal boundary is unclear, the peritoneum is cut between the left sacral ligament and the left anterior wall of the rectum, and then extended to the right. Both of the above 2 methods can effectively avoid injury to the intestine. The peritoneum on the surface of the rectum is opened, the rectum is gently pushed down, the posterior of the vagina is fully freed, and at the same time an assistant uses a vaginal separator to push the posterior wall of the vagina (Fig. 1). The separator is used to smoothly push the rectovaginal space open, expose the posterior wall of the vagina, and is slide to the middle of the vagina (about 4–5 cm from the vaginal opening). Further separation is performed under microscopic visualization, and the posterior wall of the vagina is incised transversely inside of the left and right sacral ligaments (Fig. 2). The extraction bag is then inserted through the vaginal incision (Fig. 3).

**Fig. 4:**
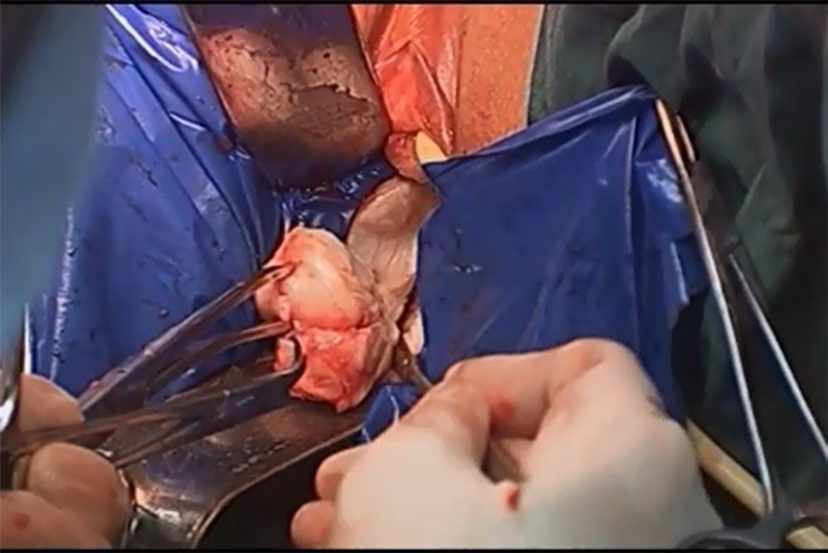
The myoma tissue is taken out of the bag with peeling to reduce the size of the tissue specimens

**Fig. 5:**
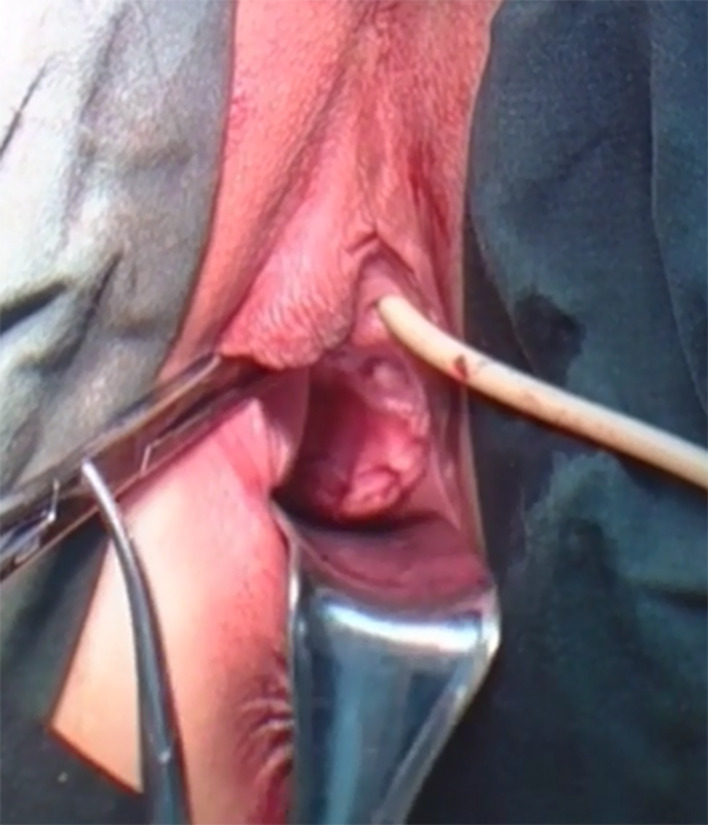
The sutured vaginal incision

All myoma tissue is placed into the bag, and the top of the bag is tied. The other end of the bag is opened and fixed to the vulva to protect the vulva from contamination. The myoma tissue is then removed through the opening of the bag. If the myoma is large, it can be “apple peeled” under direct vision at the vaginal outlet to reduce the size of the specimens and remove the tissue (Fig. 4). A gauze roll is then inserted into the vagina to prevent air leakage. A “0” absorbable suture is used to suture the open peritoneum over the uterus and rectum, the original anatomical structure is restored, and the abdominal cavity is irrigated. The pneumoperitoneum is released, and the vaginal wall mucosa is closed with 2 − 0 absorbable suture (Fig. 5). The abdominal incisions are closed with 1 − 0 absorbable suture. Iodophor gauze is placed in the vagina for completion of the operation.

### Perioperative management

All women received intravenous prophylactic antibiotics before the skin incision, and the antibiotics were continued until 24 h after surgery. Sufentail, 5 µg, was given 30 min before the end of the operation. Postoperatively, parecoxib sodium injection was given twice every 12 h. On postoperative day 1, the urinary catheter and the vaginal tamponade were removed. If the patient’s temperature is normal 2 days after the operation, she is discharged. Patients are instructed to refrain from intercourse for 1 month, and are seen in the outpatient department 1 month after surgery for follow-up.

### Statistical analysis

Descriptive statistics were used to describe patient clinical and demographic characteristics, and surgical data. Continuous variables were expressed as mean and standard deviation, or median and interquartile range, based on the distribution. Categorical variables were expressed as frequency and percentage. A value of p < 0.05 was considered to indicate statistical significance. All statistical analyses were performed using SPSS version 23.0 software.

**Table 1:** General data of patients

Variables	Study cohort (n = 511) Mean ± SD
Age, yrs	40.82 ± 5.86
Body mass index, kg/m^2^	23.84 ± 3.87
Parity	
0	60(11.74)
1	393(76.91)
≥2	58(11.35)
Number of myomas	
1	99(19.37)
2	107(20.94)
3	153(29.94)
＞3	152(29.75)
Myoma diameter, cm	8.44±3.56(5–25)
Myomas ≥ 10cm and < 12 cm	36(7.05)
Myomas ≥ 12 cm	15(2.94)
Indication for surgery	
Abnormal uterine bleeding	376(73.58)
Pain	60(11.74)
Subfertility/infertility	21(4.11)
frequency of urinatior	53(10.37)
abnormal defecation	1(0.2)
History of pelvic surgery	
Yes	231(45.21)
No	280(54.79)
Specimen weight, g	789.23±276.97
Medical complications*	
Yes	153(29.94)
No	358(70.06)

* Medical complications include hypertension, diabetes, anemia hemoglobin < 100 g/L, symptomatic heart disease, pulmonary disease, kidney disease, and abnormal thyroid function

**Table 2:** Intra- and postoperative details

Characteristics of the surgery and the postoperative outcomes	Study cohort (n = 511)
Operative time, min	129.01±53.13
transvaginal Specimen time,min	6.01±4.5
Vaginal incision suture time,min	8.13±2.6
Estimated blood loss,mL	175.99±210.96
Blood transfusions	9(1.76)
Fever	17(3.33)
Hospital stay, d	2.1±0.6
Intraoperative complications	13(2.54)
Early postoperative complications	0
Postoperative pain score (6 hours)	1.12±0.32
Postoperative pain score (24 hours)	0.76±0.11

## Results

During the study period 923 laparoscopic myomectomies were performed, and the data of 511 were included and analyzed in this study. Cases not included in this study included those with an expanded umbilical incision or morcellation in the protective bag, adhesions preventing exposure of the Douglas cavity, and refusal to have the tissue removed though a vaginal incision.

The characteristics of patients included in the analysis are summarized in Table 1. The mean age of the patients was 40.82 ± 5.86 years, mean BMI was 23.84 ± 3.87 kg/m^2^, and 60 women were nulliparous. The indications for myomectomy included abnormal uterine bleeding (n = 376), pain (n = 60), and subfertility/infertility (n = 21). The mean largest myoma diameter was 8.44 ± 3.56 cm (36 myomas ≥ 10 cm and < 12 cm; 15 myomas ≥ 12 cm), and the mean myoma weight was 789.23 ± 276.97 g. Of the patients, 231 had a history of Cesarean section or other pelvic surgery. There were no conversions to open surgery.

The mean operative time was 129.01 ± 53.13 min, and the mean intraoperative blood loss was 175.99 ± 210.96 mL. There were 13 intraoperative complications; all were an intraoperative blood loss > 500 mL, and 9 patients required a transfusion. Postoperatively, 17 women were febrile. There were no injuries of the bowel, bladder, major vessels, or other organs. There were no ruptures of the tissue extraction bags. The mean hospital stay was 2.1 ± 0.6 days (Table 2).

Postoperative follow-up 30 days after surgery showed all of the vaginal incisions had healed well, and there were no cases of infection or vaginal bleeding. No incisional hernias, pelvic infections, or vaginal adhesions were noted during follow-up for 3 months after the operation. A total of 238 women participated in a sexual life questionnaire survey 6 months after the operation. The female sexual function index questionnaire (FSFI) was used to evaluate the sexual function of patients, and there was no statistically significant difference pre-operation.

Postoperative pathological examination of the tissue specimens found 499 cases of uterine leiomyoma, 7 cases of cellular uterine leiomyoma,1 case of fatty degeneration of leiomyoma, 1 case of hyaline degeneration, 1 case of venous leiomyoma, 1 case of low-grade endometrial stromal sarcoma, and 1 case of leiomyosarcoma. The patients with sarcoma and low-grade endometrial stromal sarcoma subsequently underwent total hysterectomy and bilateral adnexectomy. Currently, at 2.5 years and 4 years after surgery, respectively, no patients have had a recurrence.

Of the patients, 37 had a vaginal delivery and there were no lacerations of the posterior wall vaginal incision during delivery.

## Discussion

One of the most important steps of laparoscopic myomectomy is specimen removal. The resected myoma tissue often needs to be cut into small pieces using power morcellation before it can be extracted. However, laparoscopic morcellation increases the chances malignant or abnormal (e.g., endometriosis) being spread into the abdominal and pelvic cavity, and potential rapid progression of the disease. The recurrence rate after operation is significantly increased, and the survival rate is reduced, which affects the prognosis [16–18]. Since the warnings regarding laparoscopic power morcellation by the US FDA on April 17 and November 2, 2014, gynecologists have been exploring new methods for specimen removal to avoid the adverse consequences of morcellation.

Currently, a sterile plastic bag is widely used as a specimen bag; the bag is placed into the abdomen, the tissue sample is placed into the bag, the morcellation is performed on the tissue in the bag, and then the bag with the tissue is removed [19–24]. This method minimizes the potential risk of disseminating malignant cells. However, it does not completely eliminate the risk of spreading malignant cells, and there is still the possibility of tumor spread caused by bag rupture, and cell contamination of the light source lens during the bag rotation process [25].

In our experience, it takes longer to perform morcellation of a myoma in a protective bag than it does to surgically remove the myoma and close the myoma space. In addition, specimen retrieval devices usually require a 10- or 15-mm trocar to be inserted into the abdomen. The enlargement of skin and fascial incisions to allow the use of the required size trocars can increase the risk of port site complications, such as an incisional hernia [26, 27]. Extracting the specimen through an incision in the posterior vaginal wall save a lot of time due to the elastic nature of the vaginal wall. In addition, not increasing the size of any of the abdominal incisions reduces the changes of an incisional hernia.

Removing the tissue specimen through an abdominal wall incision or umbilical incision that is extended to 3–4 cm has been reported [28, 29]. This method avoids the use of morcellation, but increases the potential of incisional complications and affects postoperative cosmesis. In addition, a larger incision is associated with a greater degree of postoperative pain.

Removing a tissue specimen obtained laparoscopically through the vagina has been reported previously [30–33]. However, most reports describe an incision in the posterior fornix, which is at a high position and making and suturing the incision is difficult. Lagana et al. [15] reported 692 cases of laparoscopic myomectomy and removal of specimens through an incision in the posterior fornix of the vagina, and this is the largest reported case series of this technique. No infections at the level of the colpotomy or pelvic infections, the greatest concerns when using transvaginal extraction for specimen retrieval, were noted within 30 days from surgery. However, the maximum diameter of the myoma removed was a mean of 6.64 ± 2.2 cm, and there was no standardized functional questionnaire administered to patients postoperatively, and there was no long-term follow-up, including the rate of vaginal delivery.

Based on the descriptions of prior techniques of specimen retrieval through the vagina, that is, extracting surgical specimens through an incision in the middle of the posterior wall of the vagina under the protection of a specimen bag. The vaginal incision in our method is made in the middle section of the vagina, about 5 cm away from the external opening of the vagina, and thus has no impact on pelvic floor structures. The sample bag is placed into the abdomen through the vaginal incision, the specimens are placed in the bag, and the top of the bag is tied. The other end of the bag is fixed to the vulva and opened and the tissue is removed. The tissue specimens are never in direct contact with the vaginal incision during the whole process, so as to reduce the occurrence of postoperative incisional infection and pelvic inflammation. The myoma specimen does not require morcellation, and thus risks related to morcellation are avoided. In addition, we use the specially made extraction bag with marks. The specimen(s) are cut into smaller pieces while in the extraction bag with a scalpel, cold knife, which is not easy to cause side injury, and no residual specimen tissue is left in the pelvic or abdominal cavity.

Based on our surgical experience, as long as the minimum diameter of the myoma is less than 8 cm, the specimen can be taken out through a transverse vaginal incision. The position of the incision in the vaginal wall is relatively low, and the specimen can be placed in the true pelvis, which is conducive to the removal of larger specimens. If the specimen cannot be completely removed though the incision due to its size, the volume can be reduced under direct vision using the “apple peeling” method. This incision in the posterior vaginal wall is not as deep as a posterior fornix incision, and the posterior wall tissue of the vagina is more spacious and elastic than the posterior fornix, which is more conducive to the removal of large myoma. After specimen removal, an incision in the posterior vaginal wall is relatively easy to suture. The method does not require enlargement of any of the abdominal incisions, and thus does not increase the risk of an incisional hernia. Helgstrand et al. [34] reported that the incidence of incisional hernia when a 5 mm incision is used is far lower than when the incision is larger.

Transvaginal surgery is a method that is familiar to almost every gynecologist. When cutting the vagina, the method of “up and down clamping” is used. As long as the rectal attachment behind the vagina is fully separated, lift up the posterior wall of the vagina using a posterior wall separator (if there is no such device, it can be replaced by the vaginal gauze roll). The vaginal incision does not need to be sutured under Laparoscopic, which reduces the difficulty of operation and is easy to master.

Although transvaginal surgery is a common method of gynecological surgery, gynecologists still maintain a cautious attitude towards transvaginal specimen collection. First, there is the concern that transvaginal specimen collection increase the risk of pelvic/abdominal infections. Several authors have inferred conclusions on the potential risk of infections with transvaginal specimen retrieval from studies of hysterectomy. Surgical site infection after vaginal hysterectomy is rare (1%) [35], lending support that the risk of infection after transvaginal specimen retrieval is low. Notably, the surgical field in vaginal hysterectomy is exposed to the vaginal flora during the entire operation, and surgical and instrument manipulations are carried via the vagina throughout the procedure. With laparoscopic myomectomy, the time the incision in the vagina is open is usually short, and the incision is made late in the operation. Thus, potential magnitude of contamination would seem to be low. In addition, the positive pressure between the peritoneal cavity and the vagina generated by the pneumoperitoneum may reduce the risk of peritoneal bacterial contamination. Our results of 511 patients showed no postoperative pelvic infections.

Another concern with our method is if the scar of a vaginal incision can affect the sexual life of patients. A recent study demonstrated that dyspareunia was not observed during the follow-up period in 75 patients who underwent laparoscopic myomectomies with transvaginal specimen retrieval [36]. Similarly, no complaints of dyspareunia were reported at the 30-day follow-up in a retrospective analysis of 316 women who underwent transvaginal specimen extractions with enclosed manual morcellation following laparoscopic myomectomies [37]. There was no statistically significant difference between our patients’ sexual function questionnaire and preoperative questionnaire [38]. In addition, from an anatomical perspective an incision located in the middle of the vagina is not a stress-point of sexual intercourse. Most of the vaginal nerves are distributed on both sides of the fornix, the anterior wall of the vagina, and the distal end of the vaginal wall. There are only sparse nerves in the posterior wall of the vagina [39], and it is rare to damage vaginal nerves due to an incision of the middle section of the posterior wall of the vagina.Thirdly, whether the retroperitoneal site is adhered further leads to infertility. The pelvic peritoneal incision and vaginal wall incision are not at the same level, and they are sutured separately to restore the original anatomical structure. This can reduce the risk of pelvic adhesions caused by an incisional inflammatory reaction. Colpotomy for peritoneal access has been proven to be safe from large case series in the gynecological and non-gynecological literature, and no significant sequelae on sexual function and fertility have been reported [40]. At second-look laparoscopy, Nezhat et al. [41] noted no adhesions in the cul-de-sac of 22 women who had undergone laparoscopic posterior colpotomy at initial operative laparoscopy.

Finally, there is no relevant literature regarding the effect of an incision in the posterior vaginal wall on vaginal delivery. Of the patients we followed-up, there were 83 pregnancies, of which 37 were delivered vaginal without any vaginal lacerations.

Based on our study, we can draw a number of conclusions. (1) The time required to remove a specimen through the vagina is short. Myoma with a diameter of < 8 cm can be directly and completely removed through a vaginal incision, which avoids time consuming morcellation. For the myoma with a diameter > 8 cm, because the vaginal incision is closer to the vaginal opening it is easier to reduce the volume by the “apple peel” method, and thus remove the specimen. (2) Transvaginal specimen removal avoids extending abdominal wall incisions, and thus does not increase the risk of an incision hernia, does not increase postoperative pain, and does not affect cosmetic outcomes. (3) The satisfaction of patients with transvaginal specimen removal is high, and postoperative pain is significantly reduced. There are only sparse nerves in the posterior wall of the vagina, and thus postoperative pain from an incision in the posterior wall is not great [42]. (4) The method avoids the use of morcellation and thus should not increase the risk of spreading malignant cells in the abdominal and pelvic cavities.

There are also limitations of transvaginal specimen removal. Patients who are not sexually active and those who are adverse to vaginal surgery may refuse the procedure. In addition, patients with endometriosis or pelvic inflammation resulting in closure of the Douglas cavity are not eligible for this surgical approach [15, 43].

The incision and opening of the posterior vaginal space should be carefully performed, exposing the Douglas cavity. The peritoneum is cut at the junction between the rectum and the vaginal wall, and the incision length should not exceed the space between the bilateral sacral ligaments. An assistant uses a vaginal separator to hold the posterior wall of the vagina, and the rectovaginal space can be successfully pushed open, effectively avoiding injury to the intestine. For patients with adhesions there is a risk of intestinal injury [44]. The method should be avoided in patients with complete closure of the Douglas cavity.

This study has several strengths. First, the large number of cases is a major strength of the study. Second, all procedures were performed by the same medical team, and all postoperative vaginal examinations were performed by the chief surgeon. Third, transvaginal surgery is familiar to most gynecologist, and method is easy to learn and master. Fourth, we have developed a vaginal separator, which is easy to operate, more improve the safety of surgery, shorten the time of vaginal posterior wall incision, and reduce the difficulty of surgery; At the same time, we use a self-made, bright colored extraction bag, and the bright color makes it visible compared to the surrounding tissue which helps to avoid cutting the bag or injuring surrounding organs.

However, this study has some limitations that should be considered. First, this was a single-center, retrospective study. In addition, there was not control group for comparison.

## Conclusion

Our results indicate that an incision of posterior wall of the vagina and in-bag transvaginal extraction is a feasible option for surgical specimen retrieval after laparoscopic myomectomy, and has the potential to reduce the risk of the spread of malignant cells.

## Data Availability

The original contributions presented in the study are included in the article/supplementary materials, and further inquiries can be directed to the corresponding author. All methods were performed in accordance with relevant guidelines and regulations.
